# Comparison of pharmaceutical, illicit drug, alcohol, nicotine and caffeine levels in wastewater with sale, seizure and consumption data for 8 European cities

**DOI:** 10.1186/s12889-016-3686-5

**Published:** 2016-10-01

**Authors:** Jose Antonio Baz-Lomba, Stefania Salvatore, Emma Gracia-Lor, Richard Bade, Sara Castiglioni, Erika Castrignanò, Ana Causanilles, Felix Hernandez, Barbara Kasprzyk-Hordern, Juliet Kinyua, Ann-Kathrin McCall, Alexander van Nuijs, Christoph Ort, Benedek G. Plósz, Pedram Ramin, Malcolm Reid, Nikolaos I. Rousis, Yeonsuk Ryu, Pim de Voogt, Jorgen Bramness, Kevin Thomas

**Affiliations:** 1 Norwegian Institute for Water Research (NIVA), Gaustadalléen 21, Oslo, NO-0349 Norway; 2 Norwegian Centre for Addiction Research, Faculty of Medicine, University of Oslo, PO box 1078, Blindern, Oslo 0316 Norway; 3 IRCCS-Istituto di Recerche Farmacologiche “Mario Negri”, Via La Masa 19, Milan, 20156 Italy; 4 Research Institute for Pesticides and Water, University Jaume I, Avda. Sos Baynat s/n, Castellón, E-12071 Spain; 5 Department of Chemistry, University of Bath, Faculty of Science, Bath, BA2 7AY UK; 6 KWR Watercycle Research Institute, Chemical Water Quality and Health, P.O. Box 1072, Nieuwegein, 3430 BB The Netherlands; 7 Department of Pharmaceutical Sciences, Toxicological Center, Campus Drie Eiken, University of Antwerp, Universiteitsplein 1, Antwerp, 2610 Belgium; 8 Eawag, Swiss Federal Institute of Aquatic Science and Technology, Dübendorf, CH-8600 Switzerland; 9 Department of Environmental Engineering, Technical University of Denmark, Miljøvej, Building 115, Kgs. Lyngby, DK-2800 Denmark

**Keywords:** Wastewater-based epidemiology, Drug consumption, Correlation, Europe-wide study, Seizures, Sales statistics

## Abstract

**Background:**

Monitoring the scale of pharmaceuticals, illicit and licit drugs consumption is important to assess the needs of law enforcement and public health, and provides more information about the different trends within different countries. Community drug use patterns are usually described by national surveys, sales and seizure data. Wastewater-based epidemiology (WBE) has been shown to be a reliable approach complementing such surveys.

**Method:**

This study aims to compare and correlate the consumption estimates of pharmaceuticals, illicit drugs, alcohol, nicotine and caffeine from wastewater analysis and other sources of information. Wastewater samples were collected in 2015 from 8 different European cities over a one week period, representing a population of approximately 5 million people. Published pharmaceutical sale, illicit drug seizure and alcohol, tobacco and caffeine use data were used for the comparison.

**Results:**

High agreement was found between wastewater and other data sources for pharmaceuticals and cocaine, whereas amphetamines, alcohol and caffeine showed a moderate correlation. methamphetamine and 3,4-methylenedioxymethamphetamine (MDMA) and nicotine did not correlate with other sources of data. Most of the poor correlations were explained as part of the uncertainties related with the use estimates and were improved with other complementary sources of data.

**Conclusions:**

This work confirms the promising future of WBE as a complementary approach to obtain a more accurate picture of substance use situation within different communities. Our findings suggest further improvements to reduce the uncertainties associated with both sources of information in order to make the data more comparable.

**Electronic supplementary material:**

The online version of this article (doi:10.1186/s12889-016-3686-5) contains supplementary material, which is available to authorized users.

## Background

The chemical analysis of the exogenous biomarkers of drug consumption in wastewater has been shown to be an interesting approach for studying drug use within a defined population [1]. The approach, termed wastewater-based epidemiology (WBE), was first applied in 2008 to study illicit drug use in three Italian cities [2] and it has since been extended to a wider range of exogenous biomarkers specific to the use of alcohol [3–5], tobacco [6], caffeine [7] and selected pharmaceuticals [8, 9]. To date there has been very limited focus on evaluating the relationship between WBE data to other drug use measures.

The use of pharmaceuticals in society is usually determined by prescription data or self-reports. However, pharmaceutical prescription or sales data involve several sources of uncertainties such as the inappropriate use of a medication, household disposal through the toilet or sink, sales without prescription or illegal acquisition [10–12]. Self-reports on adherence are potentially subject to recall and reporter bias [13]. An alternative could be to quantitatively measure pharmaceuticals in wastewater as an indicator of use [14–16].

Europe has a significant market for illicit drugs sustained by both domestic production and import from other regions [17]. Different monitoring approaches have been used to investigate the scale of drug use. Police and custom’s seizures, population surveys, hospital admissions, drug-overdose deaths or treatment programs have been the most reliable data for drug use estimates [18–20]. Under-reporting is still apparent despite the use of a number of different sources of information to improve the level of accuracy of self-reporting [21]. Seizure data may not give the correct picture and surveys suffer from reporting biases and low response rates [22]. WBE has been introduced as a promising alternative or addition to these sources of data [23–25].

There is also a need to be able to accurately determine the consumption of licit drugs, such as alcohol, nicotine and caffeine. Alcohol consumption can be estimated from sales statistics but trafficking, illegal production, stock piling or waste can affect such data. Reid and co-workers for the first time applied WBE to obtain complementary information on community alcohol consumption by analysing the alcohol metabolite, ethyl sulfate (EtS) in wastewater [4]. This approach has since been used in other studies to report the rates of alcohol consumption in populations [3, 26], but the value of these biomarkers has yet to be determined. Tobacco use is commonly deduced from tobacco sales statistics. Prevalence surveys and the aggregate between production and trade statistics can also yield the total consumption estimates [27]. However, these data may bias the total consumption estimates (similar to alcohol) indicating that additional methods are required in order to improve tobacco use estimations. WBE has also been used to determine nicotine use through the measurement of its residues in wastewater at national level [6, 7, 28]. These data were compared with local tobacco sales statistics finding good agreement [29]. Caffeine is present in coffee and tea as well as other products such as energy drinks, chocolate and certain medicines. Caffeine occurrence has been investigated to assess its patterns of use across different communities [7, 30].

Very few studies to date have correlated wastewater data with other sources of data. So far, pharmaceutical concentrations in wastewater have been related to the amounts used in a population by using prescription data [11, 31–33]. A Spanish study compared wastewater concentrations of 17 pharmaceuticals with annual sales data and found a good correlation [31]. The same was true for studies from Australia [32], Italy [33], and Belgium [11]. One study compared cocaine loads in wastewater in Oslo with cocaine measures from population survey data and drug tests among car drivers and found a good correlation [21]. A recent study in Belgium compared WBE results with survey data collected simultaneously in a small community of around 30,000 inhabitants for alcohol and nicotine metabolites [34]. No significant correlations were found, but the study was impaired by a typically low response to the surveys. Discrepancies between wastewater and national survey data have also been found in Australia [35].

The present study aims to compare WBE data with other sources of drug use data in order to assess its correlation. The study uses concentrations of pharmaceuticals, illicit drugs, alcohol, nicotine and caffeine in wastewater samples from different European cities and compares these with other sources of data for drug use. The data used for comparison were pharmaceutical prescriptions (morphine, methadone, atenolol, metoprolol, methylphenidate, carbamazepine, citalopram, paracetamol, oxazepam and diclofenac), police drug seizure data for illicit drugs (cocaine, amphetamine, and MDMA (ecstasy)), and consumption data for alcohol, tobacco and caffeine metabolites (EtS; cotinine, nicotine and hydroxycotinine; caffeine, paraxanthine, 1-methylxanthine, 7-methylxanthine, 1-methyluric acid and 1,7-dimethyluric acid).

## Methods

### Wastewater samples

Twenty-four hour composite inlet sewage samples were collected from 8 different European wastewater treatment plants (WWTP) over 7 consecutive days in March 2015. All samples were stored in plastic containers, immediately frozen at −20 °C to prevent degradation of the urinary metabolites and sent to Oslo and Milan within 24 h in cool boxes with dry ice or ice packs to keep the samples frozen. The average wastewater flow rate (litres per day (L/day)) through each of the treatment plants was recorded for each of the daily samples. Samples were collected every morning between 07:00 and 8:30 except for Zurich where it was performed at 00:00 (see sample details in Additional file 1: Table S1).

### Extraction and analysis

The analysis of the different wastewater samples was performed using three previously validated methods [4, 7, 9]. Illicit drugs and pharmaceuticals were analysed by liquid chromatography coupled to quadrupole time-of-flight mass spectrometry (LC-QTOF) as described by Baz-Lomba and co-workers [9]. Briefly, 50 ng of the isotopic labelled internal standards (ILIS) solution mix was spiked into wastewater (100 mL) and then extracted on a fully automatable solid phase extraction (SPE) (Horizon Technology, Salem, NH, USA) with HLB extraction disks (47 mm, I.D.; Horizon Technology, City, Country). 5 μL of the final eluent were injected into the LC-QTOF system. The compounds were chromatographically separated on a Waters Acquity UPLC system (Milford, MA, USA) fitted with a Acquity UPLC HSS C18 column (1.8 μm, 2.1 mm × 150 mm) (Waters, Milford, MA, USA). A Xevo G2-S Q-TOF mass spectrometer (Waters, Milford, MA USA) was used in positive ESI mode for acquisition using MS^e^, that allows both precursor and product ion data to be simultaneously acquired during a single run. Analyses were performed using an external reference (Lock-Spray™) with Leucine-enkephalin generating a reference ion in positive mode at m/z 556.2771 that was used for real-time mass corrections in order to maintain the mass accuracy.

EtS was analysed by ion-pair liquid chromatography coupled to tandem mass spectrometry (ion-pair LC-MS/MS) as described by Reid and co-workers [4]. Briefly, an aliquot (1 ml) of each sample was spiked with deuterated internal standard (EtS-d5) at 50 ng/mL and centrifuged at 20,000 g for 10 mins. The supernatant was subsequently injected into the LC-MS/MS system (Waters, Milford, MA) equipped with an Acquity UPLC BEH C8 column (1.7 μm, 2.1 mm × 50 mm) (Waters, Milford, MA, USA). Mobile phases were 7 mM dihexylammonium acetate in water and methanol prepared by adding equimolar volumes of dihexylamine and acetic acid into the solvents.

The tobacco and coffee biomarkers were analysed by liquid chromatography-tandem mass spectrometry (LC–MS/MS) (API 5500 QqQ. Applied Biosystems-Sciex, Thornhill, Ontario, Canada) [7]. Wastewater samples were filtered through 1.6 μm GF/A glass microfiber filters and 0.45 μm mixed cellulose membrane filters purchased from Whatman (Kent, UK). An aliquot (3 mL) was spiked with the mix labelled internal standard solution and loaded into the Oasis HLB SPE cartridges. For analysis, the extract was evaporated to dryness and reconstituted in 100 μL methanol-ultrapure water (20:80, v/v), centrifuged and transferred into glass vials for instrumental analysis. Finally, 1 μL of the final extract were injected in a LC–MS/MS system. Chromatographic separation was performed using a HPLC XTerra C18 column (3.5 μm, 1 mm × 100 mm) (Waters, Milford, MA, USA) and analytes were ionized using electrospray ionization in positive mode.

### Calculations

Drug consumption in the population connected to the respective WWTP was calculated by multiplying the measured concentration (C_ww_) of each compound (ng/L) by the average daily flow rate (q_day_) (m^3^/day) to obtain the daily mass loads (mg/day). Mass loads were then divided by the population served within the catchment area (P_ww_) to obtain the amount of the drug consumed per day per 1000 inhabitants expressed as population-normalised mass loads (Eq. 1). Correction factors (k_CF_) to account for the different excretion patterns were used only for pharmaceuticals. Moreover, concentration values that were < LOQ (method limit of quantification) were replaced by LOQ/2 if at least one day in the week had a concentration value above the LOQ [36].

$$ \mathrm{Population}\hbox{-} \mathrm{normalised}\kern0.5em \mathrm{mass}\kern0.5em \mathrm{loads}\kern0.5em \mathrm{in}\kern0.5em \mathrm{W}\mathrm{W}\kern0.5em =\kern0.5em \frac{C_{ww}\kern0.5em x\kern0.5em {q}_{day}\kern0.5em x\kern0.5em {k}_{CF}}{1000\kern0.5em x\kern0.5em \left({P}_{ww}/1000\right)}\kern0.5em =\kern0.5em \frac{mg/ day}{1000\kern0.5em inh} $$

- Pharmaceuticals: Wastewater analysis vs sales data

The environmental population-normalised loads for the selected pharmaceuticals in wastewater were estimated for Oslo (Norway) from the *per capita* monthly sales data from 2012 and 2013 (Norwegian Drug Wholesales statistics, Norwegian Institute of Public Health). Norway has one of the most accurate national prescription and sales systems in the world and all data are electronically available [37]. The possibility of gathering the sales data for the area of interest decreases the level of uncertainty. Other studies [31, 33] used national-based data and assumed that consumption pattern was the same for the whole national population.

VEAS, the Oslo WWTP, treats sewage for around 600,000 people of which Oslo contributes with the 70.5 % and Akershus with the 29.5 % (8 % from Asker and 21.5 % from Bærum). The monthly sales data (gathered for Oslo and Akershus) from 2012 and 2013 remain constant for all the pharmaceuticals during the course of the two years. For the calculation of the predicted loads the amount of defined daily doses (DDD) for each pharmaceutical was multiplied by the monthly turnover by dosage (TD) and divided by the number of days of each month. The measured loads for the pharmaceuticals in wastewater were in this case multiplied by the correction factors that took into account the different urinary excretion rates.

- Illicit drugs: Wastewater analysis vs drug seizures

Measured population-normalised mass loads of benzoylecgonine, total amphetamines (as a sum of amphetamine and methamphetamine) and MDMA in wastewater were compared with the seizure data available from the 2015 European Drug Report [17]. This report provided both the number and amount of seizures in 2013 (most recent data) for each substance except for Switzerland and the Netherlands. Bramness and co-workers [38] recommended the use of number of seizures for this kind of comparison since it provides a more accurate description of the drug situation rather than the amount seized which is more vulnerable to variations. The correlations for the three drugs are calculated based on the number of seizures in 2013 as recommended previously, however, further compared with the mean amount seized from 2010–2013 reported by the United Nations Office on Drugs and Crime (UNODC).

- Legal stimulant drugs: Wastewater analysis vs sales data

Three legal stimulants were measured for comparison with sales and consumption. The alcohol consumption rates measured in wastewater were compared with the latest surveillance report on alcohol and health published by the WHO (World Health Organization, 2014) [39]. The WHO reported consumption rates for the population aged 15 or older as L/year/person. Consumption rates were corrected for the entire population and then compared with the population-normalised loads of EtS measured in the wastewater.

The measured population-normalised mass loads of nicotine in wastewater were compared with tobacco sales data (presented as number of cigarettes per smoker per day) from 2014 as reported by the Tobacco Atlas (Euromonitor International Database) [40]. These data were normalised by multiplying the corresponding correction factor for the percentage of population aged 15 or older. The mass loads for nicotine measured in wastewater were calculated as suggested by Castiglioni and co-workers [6] by summing up the loads of the two nicotine main metabolites, cotinine and hydroxycotinine.

The population-normalised mass loads of caffeine and its metabolites measured in wastewater were compared with the *per capita* coffee consumption data from 2013 referred to the total dry weight of coffee consumed rather than brewed volume (more sensitive to variations from market to market) [41]. The correlation was performed with two approaches, by comparing i) the consumption data with the mass loads of caffeine, and ii) the sum of the six metabolites together. The interpretation of the wastewater results for caffeine needs to be carefully addressed since caffeine is present in many products, such as tea, energy drinks and chocolate.

### Statistics

Descriptive statistics for each data sources are presented as means. Spearman’s correlation coefficient (ρ) was used to assess the agreement between wastewater data and pharmaceutical sales data, police seizures and sales data, respectively for pharmaceuticals, illicit drugs and legal stimulants. *P* values less than 0.05 were considered statistically significant. All the analyses were performed in R, Version 3.2.2 [42].

## Results

### Pharmaceuticals

Sales data for all of the targeted pharmaceuticals during the 24 months between 2012 and 2013 remained constant in Oslo. The coefficient of variation for sales varied between 7 % (oxazepam) and 14 % (carbamazepine). All of the selected pharmaceuticals were detected in all of the wastewater samples collected from Oslo. Paracetamol, metoprolol and carbamazepine showed the highest mean population-normalised mass loads (11.2, 6.5 and 2.4 g/day/1000 inh. respectively), while methadone and morphine were present at the lowest loads (19.0 and 94.2 mg/day/1000 inh. respectively). The day-to-day variability for each of the compounds over 7 days was relatively low with some exceptions (mean relative standard deviation (RSD) = 24 %, excluding methadone, morphine and methylphenidate). Methadone and morphine loads varied considerably during the 7 days without any pattern (RSD = 61 and 53 % respectively) while methylphenidate showed a high RSD (93 %), mainly due to the increased loads at the weekend.

**Fig. 1:**
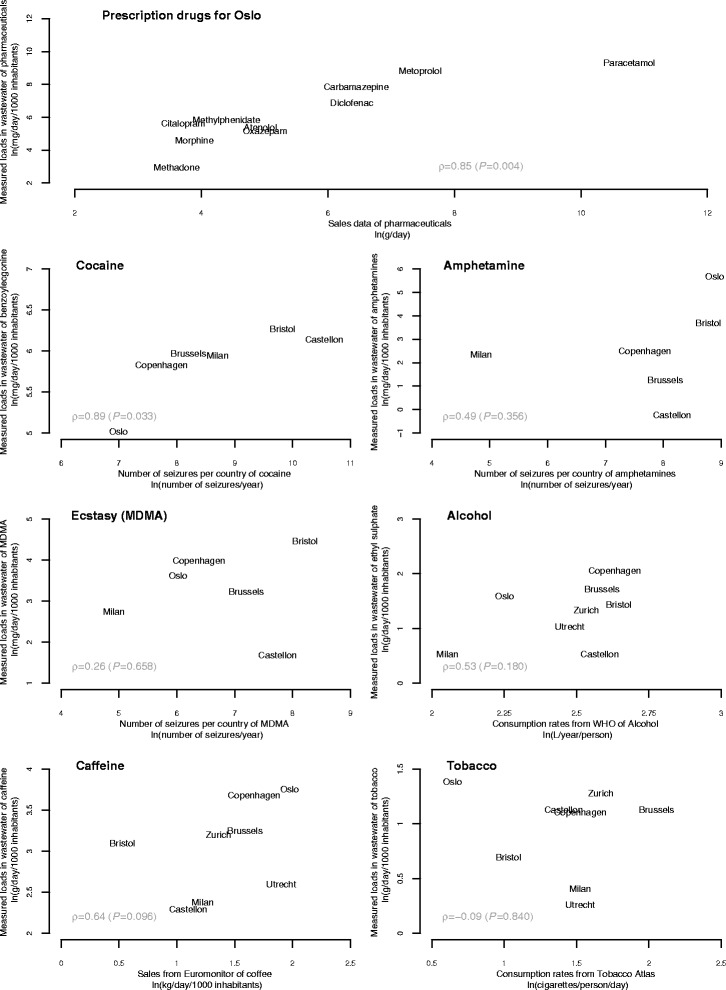
Relationship between the drug estimations from prevalence and seizures and consumption versus the population-normalised loads measured in wastewater. Spearman’s correlation coefficient (ρ) was used to assess the level of agreement between wastewater and other sources of data. P values less than 0.05 were considered statistically significant

A correction factor for the different urinary excretion rates was applied to make the data comparable with the pharmaceutical sales data for Oslo (Table 1). Sales data for all of the targeted pharmaceuticals for the 24 months between 2012 and 2013 remained constant in Oslo. The coefficient of variation for sales varied between 7 % (oxazepam) and 14 % (carbamazepine). Spearman’s rank correlation analysis was performed between the sales data in Oslo and the results obtained from the wastewater analysis for 10 different compounds showing a significant correlation (ρ = 0.85, *P* < 0.01) (Fig. 1).

**Table 1:** Summary of the monthly average sales, amount of defined daily doses (DDD) and correction factors for the different pharmaceuticals (reference)

	Sales	DDD	Correction	Loads based on sales data	Loads measured in WW
Compound	(monthly average)	(g)	factor^**a**^	(grams/day)	(mg/day/1000 inh)
Atenolol	55770	0.07	2.7 [58]	139	215.7
Carbamazepine	18920	1	6.9 [59, 58]	631	2418.5
Citalopram	61708	0.02	9.6 [58]	41	267.2
Diclofenac	18920	0.1	8.3 [60]	588	961.3
Methadone	44698	0.02	3.6 [32]	37	19.0
Methylphenidate	80586	0.03	50.0 [61]	81	323.4
Metoprolol	347042	0.15	21.5 [58]	1735	6543.8
Morphine	14718	0.03	3.1 [2]	49	94.2
Oxazepam	89610	0.05	1.7 [62]	149	168.6
Paracetamol	471511	3	2.1 [58]	47151	11231.3

^a^ Correction factor is based on the expected amount excreted in urine

Sales data average estimated over 24 months in 2012 and 2013. Population-normalised loads measured in wastewater data for pharmaceuticals in Oslo

### Illicit drugs

Benzoylecgonine was present in all of the wastewater samples with population-normalised loads ranging between 151.5 (Oslo) and 672.6 mg/day/1000 inh. (Zurich) determined. With the exception of Oslo, all of the loads showed similar values for the cities studied. The population-normalised loads for amphetamine and methamphetamine found in wastewater from Oslo were similar (122.3 and 172.4 mg/day/1000 inh., respectively). Amphetamine was also measured in Bristol and Utrecht while methamphetamine was measured in Brussels, Milan, Zurich, Copenhagen, and at very low concentrations in some of the samples from Castellon. MDMA was detected in wastewater from all locations but not in every sample. The mean daily population-normalised loads for MDMA ranged from 5.4 mg/day/1000 inh. for Castellon to 121.5 mg/day/1000 inh. for Utrecht.

The measured wastewater loads for illicit drugs were compared with national drug seizure data [17] (Table 2). The lowest and highest number of cocaine seizures and quantity seized in 2013 was in Norway and Spain respectively. The Netherlands, Belgium and Italy also reported large amounts of cocaine while the United Kingdom reported the second highest number of seizures within the studied countries. The reported seizures for amphetamine were much higher in the United Kingdom. In general all the countries reported similar values except for Italy with very low number of seizures and amount seized reported. The Netherlands and Switzerland did not report data on amphetamine and methamphetamine. In general, few seizures were reported on methamphetamine. Norway presented the highest total number of seizures in 2013 (4,210 seizures) and mean amount seized from 2010–2013 (161 kg/year). For MDMA, the United Kingdom reported the highest number of seizures in 2013 (3,716 seizures) while The Netherlands the highest mean amount seized between 2010 and 2013 (1,750,867 tablets/year).

**Table 2:** Summary of the seizures and population-normalised loads measured in wastewater data for cocaine, amphetamines and MDMA

	EMCDDA Drug Report 2015			UNODC Statistics			Wastewater Analysis 2015		
	Number of seizures in 2013			Amount seized (2010–2013)			Measured loads		
	(number/year)			(kg/year)			(mg/day/1000 inh)		
City	Cocaine	Amphetamines	MDMA	Cocaine	Amphetamines	MDMA^a^	Benzoylecgonine	Amphetamines	MDMA
Norway/Oslo	1086	7229	411	94	354	6468	151.5	294.7	37.7
Spain/Castellon	38033	3471	2301	20689	297	312780	463.9	0.8	5.4
Belgium/Brussels	3653	3085	1338	10127	197	40341	390.9	3.5	25.4
UK/Bristol	18569	6515	3716	3216	1223	494300	528.7	41.6	86.3
Netherlands/Utrecht	-	-	-	10784	807	1750867	299.5	52.9	121.5
Italy/Milan	6031	128	136	5113	41	26311	380.9	10.4	15.5
Switzerland/Zurich	-	-	-	307	40	23354	672.6	32.0	64.8
Denmark/Copenhagen	2286	2167	590	205	275	35430	337.2	11.7	53.1

^a^ Number of tablets per year

Correlation analysis was performed between wastewater data measured in 2015 and the number of seizures reported in 2013. The comparison for the illegal drugs was performed using the most recent data available (2015 European Drug Report with data from 2013 [17]). As for pharmaceuticals, there was a time mismatch between both datasets, however, seizure trends remained relatively stable during the last years. A significant positive correlation was found between the measured loads of benzoylecgonine and the number of seizures of cocaine reported by the different countries (ρ = 0.89, *P* = 0.03), while non-significant correlation was found between wastewater results and the number of seizures for amphetamines (ρ = 0.49, *P* = 0.36) and MDMA (ρ = 0.26, *P* = 0.66). Wastewater data were also compared with the mean amount seized from 2010–2013 reported by the UNODC, but non-significant correlation was found between the amount seized and the wastewater data for cocaine, amphetamines and MDMA.

### Alcohol, nicotine and caffeine

Table 3 shows the measured population-normalised mass loads, sales and consumption data for the three legal drugs studied; alcohol, nicotine and caffeine. Copenhagen, Brussels and Oslo showed the highest EtS population-normalised mass loads in wastewater (7.7, 5.6 and 4.9 g/day/1000 inh. respectively) while Castellon and Milan showed the lowest level of alcohol use during the wastewater study period (both with 1.7 g/day/1000 inh). The reported WHO alcohol consumption rates were very similar for all the cities within a range of 11.9–14.1 L/year/person except for Italy and Norway, which showed lower levels (7.8 and 9.5 L/year/person respectively). Spearman’s rank correlation analysis showed, however no-correlation between the two data sources (ρ = 0.53, *P* = 0.18).

**Table 3:** Summary of consumption estimates and population-normalised loads measured in wastewater data for alcohol, tobacco and caffeine

	Alcohol		Tobacco		Caffeine (Coffee)		
	Reported	Wastewater	Reported	Wastewater	Reported	Wastewater	
	WHO	EtS	Euromonitor Int.	HydCot + Cot	Euromonitor Int.	Caffeine	Sum Metabolites
City/Country	(L/year/person.)	c(g/day/1000 inh)	(cigarettes/person/day)	(g/day/1000 inh.)	(kg per capita in 2013.)	g/day/1000 inh	
Oslo/Norway	9.5	4.9	1.5	6.7	7.2	42.9	129.7
Castellon/Spain	13.2	1.7	4.8	5.2	3.0	9.9	74.2
Brussels/Belgium	13.3	5.6	4.0	5.3	4.9	25.8	116.6
Bristol/UK	14.1	4.2	2.1	3.4	1.7	22.1	112.8
Utrecht/Netherlands	11.9	2.8	2.2	2.1	6.7	13.4	57.3
Milan/Italy	7.8	1.7	4.0	2.6	3.4	10.8	49.9
Zurich/Switzerland	12.6	3.8	4.7	6.0	3.9	24.5	141.0
Copenhagen/Denmark	13.9	7.7	3.9	2.1	5.3	39.4	154.1

The measured load profiles of nicotine in wastewater varied among the 8 cities. The measurements in Oslo showed the highest mass loads level with 4.0 g/day/1000 inh. followed by Zurich, Brussels and Castellon with 3.6, 3.1 and 3.1 g/day/1000 inh. respectively. Milan and Utrecht showed the lowest population-normalised mass load rates (1.5 and 1.3 g/day/1000 inh. respectively). These data have been compared with tobacco consumption estimates provided by The Tobacco Atlas [40]. The Tobacco Atlas reports Belgium as the country with the highest consumption rate of cigarettes (7.8 cigarettes/person/day) whereas Norway is reported as the country with the lowest rate (1.9 cigarettes/person/day). However, our analysis showed a non-significant correlation between the two data sources (ρ = −0.09, *P* = 0.84).

Oslo had the highest measured population-normalised loads for caffeine with a value of 42.9 g/day/1000 inh. followed by Copenhagen and Brussels with 39.4 and 25.8 g/day/1000 inh. respectively. By summing up all the caffeine metabolites Oslo still had a high mass load rates (129.7 g/day/1000 inh.), but Zurich and Copenhagen with 141.0 and 154.1 g/day/1000 inh. respectively were reported as the highest. There was no significant correlation between the measured loads of caffeine in wastewater and the caffeine consumption rates (ρ = 0.64, *P* = 0.09). The sum of all the caffeine metabolites measured in wastewater did not provide a better correlation (ρ = 0.33, *P* = 0.42).

## Discussion

This study compared the population-normalised mass loads of three different groups of compounds measured in wastewater with other drug use data sources. The pharmaceutical consumption was compared at local level in Oslo (Norway) while the comparison between wastewater data and other sources of data for the illicit and legal stimulant drugs was performed at international level. The wastewater data for the pharmaceuticals and also cocaine showed a high correlation with other data sources for use. For amphetamines, alcohol and caffeine a moderate, but non-significant correlation was observed, while wastewater data on MDMA and nicotine did not correlate with other sources of data.

The correlation between wastewater data for pharmaceuticals and sales data corroborates the findings of an earlier study finding good agreement between the sales data of metoprolol and cetirizine and measured loads of these drugs in wastewater in Oslo [43]. In addition, the pharmaceuticals wholesale data used in the current study were from Oslo only and extracted only for the month of interest. Hence, the accuracy may be higher than national consumption data [31–33], which does take into consideration different local patterns of use [11], or prescription databases that may miss the contributions from hospital in-patients and sales of over-the-counter drugs [44]. The current results seem to suggest that WBE data represent accurately the population drug use that is usually measured by sales data, confirming that wastewater data could be used with certainty to estimate drug use in a population.

Among the studied illicit drugs, cocaine measurements in wastewater showed the best agreement with seizure data. The wastewater results for cocaine further agree with the latest WBE multi-city study performed in Europe in 2014 in which United Kingdom, The Netherlands, Belgium, Switzerland and Spain reported the highest population-normalised loads in 2014 [45]. In the same study, Utrecht and Castellon showed the lowest national population-normalised loads of cocaine measured in wastewater for their respective countries. These national spatial differences can explain some of the discrepancies between the WBE and the national seizure data.

For all the other comparisons in the present study the relationship between wastewater data and other sources of drug use data were less than optimal. Wastewater data presents intra-country and spatial variability and the patterns of drug use also vary over the course of the year [24], while seizures and consumption data represents an annual average within the whole country. WBE results have already shown different patterns of drug use within the same country [23, 45]. For many of our analysis we had very few observations making it possible for spurious measures to influence the analysis unduly. Moreover, the different sources of data may not compare because they measure different things (E.g. number of seizures and amount seized data did not agree completely, but together may improve the general picture of the drug situation in Europe describing the main points of entrance and possible trafficking distribution). In an Australian study the wastewater data was contrary to the drug use surveys but in agreement with prevalence data, police, health agencies and the media [35]. The negative results should thus be interpreted carefully.

The sum of the amphetamine and methamphetamine measured mass loads in wastewater correlated only moderately well with the number of seizures. A contributing factor to the low agreement may be that amphetamines have a more defined “geographical drug-use patterns” than cocaine. While amphetamine is more usual in western Europe, methamphetamine use is mainly concentrated in northern Europe, Czech Republic and Slovakia [17]. This is corroborated by previous WBE studies [23, 24, 38, 43, 46] and prevalence data [17].

No relationship was observed between WBE data for MDMA and the number of seizures. Despite this, wastewater results seem to be in agreement with other studies. For example, Utrecht and Bristol showed the highest population-normalised WBE loads of MDMA which is in harmony with survey data from other sources [17]. MDMA is however a drug used more typically on special occasions and as a “party drug”, making it more sensitive to timing of WBE [47]. Also, the MDMA market has changed dramatically during the last couple of years [17] and our poor results may have been worsened by the fact that we compared wastewater results from 2015 with seizures data from 2013. Furthermore, we only had six observations due to lack of seizure data from some countries [24].

Only a moderate correlation was found between EtS loads in wastewater and WHO sales data corroborating other studies finding similar discrepancies. A study from Santiago de Compostela and Milan estimated alcohol use on wastewater data and found results 39 and 68 % lower than the estimates reported by the WHO [3]. A Belgian study found that WBE data for alcohol consumption were in agreement with values reported by the Belgian Scientific Institute of Public Health, but much lower than those reported by the WHO [5]. Lastly, a multi-city international study of alcohol consumption for 20 different cities showed that a significant correlation was found for only 11 cities between the WHO estimations and the wastewater measurements [48]. According to these studies the main limitations are related to suboptimal records over alcohol sales in many countries. The strict Norwegian government control over alcohol sales results in accurate sales statistics [4]. Still, the unregistered consumption of alcohol increased to around 26 % in 2012 which would explain the high alcohol loads measured in wastewater according to what expected by WHO [49]. In a previous study in Oslo, the alcohol use estimates obtained from WBE and those reported by the WHO were in good agreement [4]. This indicates that WBE could be a suitable marker for alcohol consumption even with the observed moderate correlation with consumption data.

No relationship was found between wastewater loads for nicotine and cigarette sales data. Here Oslo appeared to be an outlier. The popularity of dipping tobacco in Norway has been shown to have risen between 2010–2015, reaching the same prevalence levels as cigarettes (10 % against the 13 % for cigarettes) [50]. The levels of cotinine excreted from dipping tobacco users are in the same range than cigarettes [51] which would induce an overestimation when comparing wastewater with cigarettes consumption data. In addition, a large amount of dipping tobacco is disposed of into urinals and toilets resulting in increased nicotine residues in Oslo´s wastewater influent. Cigarette consumption may vary greatly within countries [40] and other studies have shown a better agreement when comparing WBE with smoking prevalence data at regional level [6, 7, 29, 52]. Nicotine metabolites have been proposed and used as quantitative biomarkers to estimate population size at local level [7] although the presented results and previous studies [53] demonstrate that the different tobacco culture-dependent and geographic behaviour may change between countries. Therefore, nicotine metabolites should not be used as a population biomarker when comparing data across different countries

Coffee consumption data and the loads of the main caffeine metabolites in wastewater showed a moderate correlation between the two data sources. There was, however, a decent relationship when the observations from Bristol were removed from the calculations. The high WBE loads of caffeine measured in Bristol could be related to the by far highest tea [54], chocolate [55] and caffeinated energy drinks consumption (also in Spain [56]). We compared the consumption data with a sum of all the caffeine metabolites resulting in an even lower agreement. Other studies have also found that metabolite excretion profiles of caffeine disagree with WBE [7]. This may have different explanations, one being large variations in caffeine content between different coffee crops [57].

As described above, there are still a large number of limitations for both types of epidemiological data. A precise ecological approach would require data gathered from the same population and at the same time, but the greatly different time-scales involved in both approaches and the logistics of sampling make this a very challenging task.

Despite the relatively good agreement with the other sources of data, it is possible that wastewater data may be typified by low temporal representativeness and high spatial variability due to different drug use and availability trends from location to location and over time and therefore, WBE results need to be carefully interpreted. Thus far, coverage of most of the WBE studies performed is typically limited to a one-week sampling program, however, for the future more extensive random-stratified sampling schemes are recommended to increase the representativeness and decrease the level of uncertainty.

Furthermore, because WBE data is an aggregate of the consumption of all segments of the population, they do not provide an easy means of assessing the importance of sex, age or socio-economical information on the findings.

The strength of WBE is however in the ability to more easily carry out longitudinal studies. Data can be provided in a more continuous flow rather than the infrequent and perhaps sporadic data-points that may be generated from traditional surveys. WBE could therefore prove to be an excellent source of data for studying the effects of police operations, education programs or public heath campaigns aimed at reducing the use of a particular drug.

Wastewater analysis and conventional epidemiological indicators are not expected to be in exact agreement, but a degree of overlap should exist and be sufficient to demonstrate the complementary character of these approaches, as demonstrated by the case studies presented in this work.

## Conclusions

A relatively good relationship between the WBE data and the other sources of information, especially for pharmaceuticals and cocaine, was found even with the limited number of cities in the present study. This study illustrates that WBE is in reality a snapshot of the drug situation in a determined location while other data often are more general, like annual prevalence or sales data, being less sensitive to quicker changes in the recent drug use patterns. Further research is needed to improve the accuracy on the consumption reports and the uncertainties associated with the WBE. The different nature of the datasets can lead to certain disagreement when comparing both sources of information. Despite this, this work has shown that WBE is currently providing complementary and valuable information to improve the general picture of the drug situation in Europe. The presented results although foreseeing a promising future, need to be interpreted carefully since they provide a rough idea about the level of agreement between WBE and other sources of data together with some interesting indicators on the current drug situation, but both sources are not exempt from uncertainties.

## Additional file

Additional file 1: Table S1. Wastewater samples information for the 8 European cities studied in 2015. The estimated population using the sewer network is showed in brackets (thousands of inhabitants within the catchment area). (DOCX 18 kb)

## Abbreviations

C_ww_: wastewater concentration
DDD: defined daily doses
EtS: ethyl sulfate
ILIS: isotopic labeled internal standards
k_CF_: correction factor
L/day: litters per day
LC–MS/MS: liquid chromatography-tandem mass spectrometry
LC-QTOF: liquid chromatography coupled to a quadrupole time-of-flight mass spectrometry
LOQ: method limit of quantification
MDMA: 3,4-methylenedioxymethamphetamine
P_ww_: population served within the catchment area
q_day_: wastewater daily flow rate
RSD: relative standard deviation
SPE: solid phase extraction
TD: monthly turnover by dosage
UNODC: United nations office on drugs and crime
WBE: wastewater-based epidemiology
WHO: World health organization
WWTP: wastewater treatment plants

## References

[1] Thomas KV, Reid MJ (2011). What else can the analysis of sewage for urinary biomarkers reveal about communities?. Environ Sci Technol.

[2] Zuccato E, Chiabrando C, Castiglioni S, Bagnati R, Fanelli R (2008). Estimating community drug abuse by wastewater analysis. Environ Health Perspect.

[3] Rodríguez-Álvarez T, Racamonde I, González-Mariño I, Borsotti A, Rodil R, Rodríguez I, Zuccato E, Quintana JB, Castiglioni S (2015). Alcohol and cocaine co-consumption in two European cities assessed by wastewater analysis. Sci Total Environ.

[4] Reid MJ, Langford KH, Mørland J, Thomas KV (2011). Analysis and Interpretation of Specific Ethanol Metabolites, Ethyl Sulfate, and Ethyl Glucuronide in Sewage Effluent for the Quantitative Measurement of Regional Alcohol Consumption. Alcohol Clin Exp Res.

[5] Boogaerts T, Covaci A, Kinyua J, Neels H, van Nuijs AL. Spatial and temporal trends in alcohol consumption in Belgian cities: A wastewater-based approach. Drug Alcohol Depend. 2016. 10.1016/j.drugalcdep.2016.01.002 26804900

[6] Castiglioni S, Senta I, Borsotti A, Davoli E, Zuccato E (2015). A novel approach for monitoring tobacco use in local communities by wastewater analysis. Tob Control.

[7] Senta I, Gracia-Lor E, Borsotti A, Zuccato E, Castiglioni S (2015). Wastewater analysis to monitor use of caffeine and nicotine and evaluation of their metabolites as biomarkers for population size assessment. Water Res.

[8] Hernández F, Ibáñez M, Bade R, Bijlsma L, Sancho JV (2014). Investigation of pharmaceuticals and illicit drugs in waters by liquid chromatography-high-resolution mass spectrometry. TrAC Trends Anal Chem.

[9] Baz-Lomba JA, Reid MJ, Thomas KV (2016). Target and suspect screening of psychoactive substances in sewage-based samples by UHPLC-QTOF. Analytica Chimica Acta.

[10] Bound JP, Voulvoulis N (2005). Household disposal of pharmaceuticals as a pathway for aquatic contamination in the United Kingdom. Environ Health Perspect.

[11] van Nuijs AL, Covaci A, Beyers H, Bervoets L, Blust R, Verpooten G, Neels H, Jorens PG (2015). Do concentrations of pharmaceuticals in sewage reflect prescription figures?. Environ Sci Pollut Res Int.

[12] Venhuis BJ, de Voogt P, Emke E, Causanilles A, Keizers PH (2014). Success of rogue online pharmacies: sewage study of sildenafil in the Netherlands. BMJ.

[13] Brier MJ, Chambless D, Gross R, Su HI, DeMichele A, Mao JJ (2015). Association between self-report adherence measures and oestrogen suppression among breast cancer survivors on aromatase inhibitors. Eur J Cancer.

[14] Ort C, Lawrence MG, Rieckermann J, Joss A (2010). Sampling for pharmaceuticals and personal care products (PPCPs) and illicit drugs in wastewater systems: are your conclusions valid? A critical review. Environ Sci Technol.

[15] Osorio V, Marcé R, Pérez S, Ginebreda A, Cortina JL, Barceló D (2012). Occurrence and modeling of pharmaceuticals on a sewage-impacted Mediterranean river and their dynamics under different hydrological conditions. Sci Total Environ.

[16] Pereira AM, Silva LJ, Meisel LM, Lino CM, Pena A (2015). Environmental impact of pharmaceuticals from Portuguese wastewaters: geographical and seasonal occurrence, removal and risk assessment. Environ Res.

[17] EMCDDA (2015). European Drug Report 2015: Trends and Developments.

[18] Kraus L, Augustin R, Frischer M, Kummler P, Uhl A, Wiessing L (2003). Estimating prevalence of problem drug use at national level in countries of the European Union and Norway. Addiction.

[19] Colpe LJ, Barker PR, Karg RS, Batts KR, Morton KB, Gfroerer JC, Stolzenberg SJ, Cunningham DB, First MB, Aldworth J (2010). The National Survey on Drug Use and Health Mental Health Surveillance Study: calibration study design and field procedures. Int J Methods Psychiatr Res.

[20] Kroutil LA, Vorburger M, Aldworth J, Colliver JD (2010). Estimated drug use based on direct questioning and open‐ended questions: responses in the 2006 National Survey on Drug Use and Health. Int J Methods Psychiatr Res.

[21] Reid MJ, Langford KH, Grung M, Gjerde H, Amundsen EJ, Morland J, Thomas KV (2012). Estimation of cocaine consumption in the community: a critical comparison of the results from three complimentary techniques. BMJ Open.

[22] Castiglioni S, Borsotti A, Riva F, Zuccato E. Illicit drug consumption estimated by wastewater analysis in different districts of Milan: A case study. Drug Alcohol Rev. 2014;35(2):128-32. 10.1111/dar.12233 25545943

[23] Ort C, van Nuijs AL, Berset JD, Bijlsma L, Castiglioni S, Covaci A, de Voogt P, Emke E, Fatta-Kassinos D, Griffiths P (2014). Spatial differences and temporal changes in illicit drug use in Europe quantified by wastewater analysis. Addiction.

[24] Thomas KV, Bijlsma L, Castiglioni S, Covaci A, Emke E, Grabic R, Hernández F, Karolak S, Kasprzyk-Hordern B, Lindberg RH (2012). Comparing illicit drug use in 19 European cities through sewage analysis. Sci Total Environ.

[25] Castiglioni S, Thomas KV, Kasprzyk-Hordern B, Vandam L, Griffiths P (2014). Testing wastewater to detect illicit drugs: state of the art, potential and research needs. Sci Total Environ.

[26] Rodríguez-Álvarez T, Rodil R, Cela R, Quintana JB (2014). Ion-pair reversed-phase liquid chromatography–quadrupole-time-of-flight and triple-quadrupole–mass spectrometry determination of ethyl sulfate in wastewater for alcohol consumption tracing. J Chromatogr A.

[27] Guindon GE, Boisclair D (2003). Past, current and future trends in tobacco use.

[28] Lopes A, Silva N, Bronze MR, Ferreira J, Morais J (2014). Analysis of cocaine and nicotine metabolites in wastewater by liquid chromatography–tandem mass spectrometry. Cross abuse index patterns on a major community. Sci Total Environ.

[29] Rodríguez-Álvarez T, Rodil R, Rico M, Cela R, Quintana JB (2014). Assessment of Local Tobacco Consumption by Liquid Chromatography–Tandem Mass Spectrometry Sewage Analysis of Nicotine and Its Metabolites, Cotinine and trans-3′-Hydroxycotinine, after Enzymatic Deconjugation. Anal Chem.

[30] Bueno MJ, Ucles S, Hernando MD, Davoli E, Fernandez-Alba AR (2011). Evaluation of selected ubiquitous contaminants in the aquatic environment and their transformation products. A pilot study of their removal from a sewage treatment plant. Water Res.

[31] Carballa M, Omil F, Lema JM (2008). Comparison of predicted and measured concentrations of selected pharmaceuticals, fragrances and hormones in Spanish sewage. Chemosphere.

[32] Lai FY, Ort C, Gartner C, Carter S, Prichard J, Kirkbride P, Bruno R, Hall W, Eaglesham G, Mueller JF (2011). Refining the estimation of illicit drug consumptions from wastewater analysis: co-analysis of prescription pharmaceuticals and uncertainty assessment. Water Res.

[33] Verlicchi P, Al Aukidy M, Jelic A, Petrović M, Barceló D (2014). Comparison of measured and predicted concentrations of selected pharmaceuticals in wastewater and surface water: A case study of a catchment area in the Po Valley (Italy). Sci Total Environ.

[34] van Wel JH, Gracia-Lor E, van Nuijs AL, Kinyua J, Salvatore S, Castiglioni S, Bramness JG, Covaci A, Van Hal G (2016). Investigation of agreement between wastewater-based epidemiology and survey data on alcohol and nicotine use in a community. Drug Alcohol Depend.

[35] Tscharke BJ, Chen C, Gerber JP, White JM (2015). Trends in stimulant use in Australia: A comparison of wastewater analysis and population surveys. Sci Total Environ.

[36] Salvatore S, Bramness JG, Reid MJ, Thomas KV, Harman C, Roislien J (2015). Wastewater-Based Epidemiology of Stimulant Drugs: Functional Data Analysis Compared to Traditional Statistical Methods. PLoS One.

[37] Norwegian Prescription Database. Available at: [http://www.norpd.no/]. Accessed April 2016.

[38] Bramness JG, Reid MJ, Solvik KF, Vindenes V (2015). Recent trends in the availability and use of amphetamine and methamphetamine in Norway. Forensic Sci Int.

[39] Organization WH. Global status report on alcohol and health-2014. Geneva, Switzerland: World Health Organization; 2014. [http://apps.who.int/iris/bitstream/10665/112736/1/9789240692763_eng.pdf?ua=1]. Accessed April 2016.

[40] Eriksen M, Mackay J, Schluger N, Gomeshtapeh FI, Drope J. The tobacco atlas, 5th edn. Georgia: The American Cancer Society, 2015. Available from: [http://3pk43x313ggr4cy0lh3tctjh.wpengine.netdnacdn.com/wp-content/uploads/2015/03/TA5_2015_WEB.pdf]. Accessed Sept 2016.

[41] Caffeine (Coffee) Consumption By Country [http://www.caffeineinformer.com/caffeine-what-the-world-drinks]. Accessed April 2016.

[42] Team RC. R: a language and environment for statistical computing, Vol 3.2.1. Vienna: R Foundation for Statistical Computing; 2013.

[43] Reid MJ, Langford KH, Mørland J, Thomas KV (2011). Quantitative assessment of time dependent drug-use trends by the analysis of drugs and related metabolites in raw sewage. Drug Alcohol Depend.

[44] Ort C, Lawrence MG, Reungoat J, Eaglesham G, Carter S, Keller J (2010). Determining the fraction of pharmaceutical residues in wastewater originating from a hospital. Water Res.

[45] EMCDDA (2015). Wastewater analysis and drugs: a European multi-city study. European Monitoring Centre for Drugs and Drug Addiction.

[46] Kankaanpää A, Ariniemi K, Heinonen M, Kuoppasalmi K, Gunnar T (2014). Use of illicit stimulant drugs in Finland: A wastewater study in ten major cities. Sci Total Environ.

[47] Harman C, Reid M, Thomas KV (2011). In situ calibration of a passive sampling device for selected illicit drugs and their metabolites in wastewater, and subsequent year-long assessment of community drug usage. Environ Sci Technol.

[48] Ryu Y, Barcelo D, Barron LP, Bijlsma L, Castiglioni S, de Voogt P, Emke E, Hernandez F, Lai FY, Lopes A, et al. Comparative measurement and quantitative risk assessment of alcohol consumption through wastewater-based epidemiology: An international study in 20 cities. Sci Total Environ. 2016. 10.1016/j.scitotenv.2016.04.138 27188267

[49] Hordvin O (2012). The Drug Situation in Norway 2012. Annual Report to the EMCDDA.

[50] Smoking habits, 2015 [https://www.ssb.no/en/helse/statistikker/royk/aar/2016-01-14#content]. Accessed April 2016.

[51] Lee PN (2014). Health risks related to dual use of cigarettes and snus – A systematic review. Regul Toxicol Pharmacol.

[52] Mackuľak T, Birošová L, Grabic R, Škubák J, Bodík I (2015). National monitoring of nicotine use in Czech and Slovak Republic based on wastewater analysis. Environ Sci Pollut Res.

[53] Chen C, Kostakis C, Gerber JP, Tscharke BJ, Irvine RJ, White JM (2014). Towards finding a population biomarker for wastewater epidemiology studies. Sci Total Environ.

[54] Where the world's biggest tea drinkers are [http://qz.com/168690/where-the-worlds-biggest-tea-drinkers-are/]. Accessed April 2016.

[55] Alberts HC, Cidell J. Chocolate Consumption, Manufacturing, and Quality in Europe and North America. Econ Chocolate. 2015;119.

[56] Zucconi S, Volpato C, Adinolfi F, Gandini E, Gentile E, Loi A, Fioriti L. Gathering consumption data on specific consumer groups of energy drinks. External Scientific Report for European Food Safety Authority. 2013;10(3):1–190.

[57] Mitchell DC, Knight CA, Hockenberry J, Teplansky R, Hartman TJ (2014). Beverage caffeine intakes in the U.S. Food Chem Toxicol.

[58] Lienert J, Güdel K, Escher BI (2007). Screening Method for Ecotoxicological Hazard Assessment of 42 Pharmaceuticals Considering Human Metabolism and Excretory Routes. Environ Sci Technol.

[59] ter Laak TL, Kooij PJ, Tolkamp H, Hofman J (2014). Different compositions of pharmaceuticals in Dutch and Belgian rivers explained by consumption patterns and treatment efficiency. Environ Sci Pollut Res Int.

[60] Moffat AC, Osselton MD, Widdop B (2011). Clarke’s analysis of drugs and poisons.

[61] Faraj BA, Israili ZH, Perel JM, Jenkins ML, Holtzman SG, Cucinell SA, Dayton PG (1974). Metabolism and disposition of methylphenidate-14C: studies in man and animals. J Pharmacol Exp Ther.

[62] Shull HJ, Wilkinson GR, Johnson R, Schenker S (1976). Normal disposition of oxazepam in acute viral hepatitis and cirrhosis. Ann Intern Med.

