# Supplementary material for: Comparison of pharmaceutical, illicit drug, alcohol, nicotine and caffeine levels in wastewater with sale, seizure and consumption data for 8 European cities
Source: BMC Public Health. 2016 Oct 1;16:1035. doi: 10.1186/s12889-016-3686-5 (PMC5045646; doi:10.1186/s12889-016-3686-5)
Supplement: Additional file 1: Table S1. — Wastewater samples information for the 8 European cities studied in 2015. The estimated population using the sewer network is showed in brackets (thousands of inhabitants within the catchment area). (DOCX 18 kb) [file 12889_2016_3686_MOESM1_ESM.docx]

SUPPLEMENTARY INFORMATION (S.I.)

**Comparison of pharmaceutical, illicit drug, alcohol, nicotine and caffeine levels in wastewater with sale, seizure and consumption data for 8 European cities**

Jose Antonio Baz-Lomba^ab^, Stefania Salvatore^b^, Emma Gracia-Lor^c^, Richard Bade^d^, Sara Castiglioni^c^, Erika Castrignanò^e^, Ana Causanilles^f^, Felix Hernandez^d^, Barbara Kasprzyk-Hordern^e^, Juliet Kinyua^g^, Ann-Kathrin McCall^h^, Alexander van Nuijs^g^, Christoph Ort^h^, Benedek G. Plósz^i^, Pedram Ramin^i^, Malcolm Reid^a^, Nikolaos I. Rousis^c^, Yeonsuk Ryu^a^, Pim de Voogt^f^, Jorgen Bramness^b^, Kevin Thomas^a^

**Corresponding author**

Jose Antonio Baz Loma: [Joseantonio.baz@niva.no](mailto:Joseantonio.baz@niva.no) Phone: 0047 98215460

**Co-authors**

Stefania Salvatore: [stefania.salvatore@medisin.uio.no](mailto:stefania.salvatore@medisin.uio.no)

Emma Gracia Lor: [emma.gracialor@marionegri.it](mailto:emma.gracialor@marionegri.it)

Richard Bade: [bade@uji.es](mailto:bade@uji.es)

Sara Castiglioni: [sara.castiglioni@marionegri.it](mailto:sara.castiglioni@marionegri.it)

Erika Castrignanò: [E.Castrignano@bath.ac.uk](mailto:E.Castrignano@bath.ac.uk)

Ana Causanilles [ana.Causanilles.Llanes@kwrwater.nl](mailto:ana.Causanilles.Llanes@kwrwater.nl)

Felix Hernandez: [hernandf@uji.es](mailto:hernandf@uji.es)

Barbara Kasprzyk-Hordern: [B.Kasprzyk-Hordern@bath.ac.uk](mailto:B.Kasprzyk-Hordern@bath.ac.uk)

Juliet Kinyua: [juliet.kinyua@uantwerpen.be](mailto:juliet.kinyua@uantwerpen.be)

Ann-Kathrin McCall: [Ann-Kathrin.McCall@eawag.ch](mailto:Ann-Kathrin.McCall@eawag.ch)

Alexander van Nuijs: [Alexander.vanNuijs@ua.ac.be](mailto:Alexander.vanNuijs@ua.ac.be)

Christoph Ort: [Christoph.Ort@eawag.ch](mailto:Christoph.Ort@eawag.ch)

Benedek G. Plósz: [beep@env.dtu.dk](mailto:beep@env.dtu.dk)

Pedram Ramin: [pear@env.dtu.dk](mailto:pear@env.dtu.dk)

Malcolm Reid: [mre@niva.no](mailto:mre@niva.no)

Nikolaos I. Rousis: [Nikolaos.Rousis@marionegri.it](mailto:Nikolaos.Rousis@marionegri.it)

Yeonsuk Ryu: [yry@niva.no](mailto:yry@niva.no)

Pim de Voogt: [w.p.devoogt@uva.nl](mailto:w.p.devoogt@uva.nl)

Jørgen Bramness: [j.g.bramness@medisin.uio.no](mailto:j.g.bramness@medisin.uio.no)

Kevin Thomas: [kth@niva.no](mailto:kth@niva.no)

Table S-1. Wastewater samples information for the 8 European cities studied in 2015. The estimated population using the sewer network is showed in brackets (thousands of inhabitants within the catchment area).

|  |  |  |  |  |  |  |  |  |
| --- | --- | --- | --- | --- | --- | --- | --- | --- |
|  | Oslo (580) | | Flow Average |  | Castellon (180) | | Flow Average |  |
|  | Date | Weekday | (m^3^ day^-1^) |  | Date | Weekday | (m^3^ day^-1^) |  |
|  | 11/03/2015 | Wednesday | 333480 |  | 25/03/2015 | Wednesday | 50228 |  |
|  | 12/03/2015 | Thursday | 308279 |  | 26/03/2015 | Thursday | 49161 |  |
|  | 13/03/2015 | Friday | 277449 |  | 27/03/2015 | Friday | 43728 |  |
|  | 14/03/2015 | Saturday | 256766 |  | 28/03/2015 | Saturday | 38301 |  |
|  | 15/03/2015 | Sunday | 250383 |  | 29/03/2015 | Sunday | 37243 |  |
|  | 16/03/2015 | Monday | 254570 |  | 30/03/2015 | Monday | 37469 |  |
|  | 17/03/2015 | Tuesday | 252721 |  | 31/03/2015 | Tuesday | 40476 |  |
|  |  |  |  |  |  |  |  |  |
|  | Brussels (950) | | Flow Average |  | Bristol (890) | | Flow Average |  |
|  | Date | Weekday | (m^3^ day^-1^) |  | Date | Weekday | (m^3^ day^-1^) |  |
|  | 18/03/2015 | Wednesday | 234264 |  | 10/03/2015 | Tuesday | 197493 |  |
|  | 19/03/2015 | Thursday | 235442 |  | 11/03/2015 | Wednesday | 204491 |  |
|  | 20/03/2015 | Friday | 234906 |  | 12/03/2015 | Thursday | 198950 |  |
|  | 21/03/2015 | Saturday | 233096 |  | 13/03/2015 | Friday | 197523 |  |
|  | 22/03/2015 | Sunday | 230375 |  | 14/03/2015 | Saturday | 252682 |  |
|  | 23/03/2015 | Monday | 234774 |  | 15/03/2015 | Sunday | 220687 |  |
|  | 24/03/2015 | Tuesday | 359951 |  | 16/03/2015 | Monday | 193194 |  |
|  |  |  |  |  |  |  |  |  |
|  | Utrecht (300) | | Flow Average |  | Milan (1120) | | Flow Average |  |
|  | Date | Weekday | (m^3^ day^-1^) |  | Date | Weekday | (m^3^ day^-1^) |  |
|  | 04/03/2015 | Wednesday | 46000 |  | 10/03/2015 | Tuesday | 423110 |  |
|  | 05/03/2015 | Thursday | 47740 |  | 11/03/2015 | Wednesday | 403240 |  |
|  | 06/03/2015 | Friday | 45030 |  | 12/03/2015 | Thursday | 412310 |  |
|  | 07/03/2015 | Saturday | 49530 |  | 13/03/2015 | Friday | 402240 |  |
|  | 08/03/2015 | Sunday | 46030 |  | 14/03/2015 | Saturday | 403020 |  |
|  | 09/03/2015 | Monday | 46900 |  | 15/03/2015 | Sunday | 422690 |  |
|  | 10/03/2015 | Tuesday | 45970 |  | 16/03/2015 | Monday | 597470 |  |
|  |  |  |  |  |  |  |  |  |
|  | Zurich (410) | | Flow Average |  | Copenhagen (530) | | Flow Average |  |
|  | Date | Weekday | (m^3^ day^-1^) |  | Date | Weekday | (m^3^ day^-1^) |  |
|  | 18/03/2015 | Wednesday | 157084 |  | 10/03/2015 | Tuesday | 148724 |  |
|  | 19/03/2015 | Thursday | 161005 |  | 11/03/2015 | Wednesday | 150936 |  |
|  | 20/03/2015 | Friday | 161427 |  | 12/03/2015 | Thursday | 147175 |  |
|  | 21/03/2015 | Saturday | 200010 |  | 13/03/2015 | Friday | 144840 |  |
|  | 22/03/2015 | Sunday | 243013 |  | 14/03/2015 | Saturday | 145197 |  |
|  | 23/03/2015 | Monday | 177167 |  | 15/03/2015 | Sunday | 137793 |  |
|  | 24/03/2015 | Tuesday | 160912 |  | 16/03/2015 | Monday | 137244 |  |
|  |  |  |  |  |  |  |  |  |
